# Editorial: The role of neurotransmitters: from normal aging to age-related diseases, volume II

**DOI:** 10.3389/fnagi.2026.2015252

**Published:** 2026-10-23

**Authors:** Yan Zhou, Xiaodan Pan, Yitong Cao, Xuntao Yin

**Affiliations:** 1 Department of Radiology, Guangzhou Women and Children's Medical Center, Guangzhou Medical University, Guangzhou, China; 2 Guangdong Provincial People's Hospital (Guangdong Academy of Medical Sciences), South China University of Technology, Guangzhou, China; 3 School of Pediatrics, Guangzhou Medical University, Guangzhou, China; 4 Department of Pediatric Rehabilitation, Guizhou Provincial Rehabilitation Hospital, Guiyang, China

**Keywords:** age-related diseases, GABA, glutamate, multimodal integration, neurotransmitters, normal aging

Neurotransmitters regulate synaptic signaling, excitatory–inhibitory balance, and large-scale network dynamics, yet their *in vivo* dynamics during aging remain difficult to quantify directly. Normal aging involves non-linear, region-specific changes in GABA, glutamate, acetylcholine, dopamine, and serotonin that frequently precede clinical symptoms and contribute to neurodegenerative and psychiatric disorders (Lee and Kim, 2022). However, the mechanistic links between molecular alterations, circuit dysfunction, and heterogeneous clinical phenotypes remain incompletely understood. Building on the first volume, this Research Topic Volume II brings together six studies spanning multiple neurotransmitter systems, disease models, imaging modalities, and computational frameworks. Rather than six isolated contributions, they converge on three cross-cutting themes: neurotransmitter imbalance as a shared pathological pathway, neuro–immune–glial crosstalk as an amplifying mechanism, and multimodal integration as a unifying framework for precision phenotyping.

The first cross-cutting theme is that neurotransmitter imbalance—not single-transmitter loss—is a shared pathway across neurological disorders. Caligiore et al. propose a system-level hypothesis in which optic nerve demyelination disrupts dopamine signaling through the lateral geniculate nucleus, superior colliculus, and striatum, promoting conversion from clinically isolated syndrome to multiple sclerosis. This model places dopamine at the intersection of visual pathway damage, basal ganglia dysfunction, and brainstem reflex abnormalities, extending dopamine beyond its classical roles in motor control and reward (Carandini et al., 2021). Chen et al. and Zhou et al. provide bibliometric analyses of glutamate and GABA in Parkinson's disease, respectively. Their findings identify basal ganglia, oxidative stress, dopamine, and α-synuclein as shared hotspots, with glutamate receptors and synaptic plasticity prominent in the glutamate-PD landscape. α-Synuclein, neuroinflammation, and anxiety emerge as frontier topics across these analyses. Together, these studies show that dopaminergic, glutamatergic, and GABA ergic imbalances converge on disrupted excitatory–inhibitory equilibrium, interacting with α-synuclein pathology and neuroinflammation to drive both motor and non-motor symptoms (Almohmadi et al., 2025).

The second cross-cutting theme is the neuro–immune–glial triad as an amplifying mechanism. Dopamine is not only a neurotransmitter but also modulates T-cell activation, cytokine release, and antigen presentation, while immune cells synthesize and respond to dopamine, as highlighted by Caligiore et al.. This bidirectional signaling suggests that focal inflammatory lesions can propagate into system-level neuronal dysfunction. Al-Sheikh et al. provide a transcriptomic resource showing that loss of the GABA-synthesizing enzyme UNC-25 in AMsh glia accelerates aging-related transcriptomic changes in ASH neurons, whereas glial-specific restoration activates Hedgehog signaling and its effector TRA-1/GLI, alongside autophagy, DNA repair, and synaptic maintenance. These findings identify glial-derived GABA as a modulator of neuronal resilience, though candidate pathways await functional validation. Liu et al. further describe how dry eye disease involves bidirectional neuroimmune signaling via dendritic cells, cytokines, neuropeptides, and diminished neurotrophic support, leading to reduced nerve density, microneuroma formation, sensory loss, and central sensitization. Collectively, these contributions demonstrate that glial and immune cells bidirectionally interact with neurons to remodel neurotransmitter availability and circuit excitability, extending neurotransmitter signaling beyond neuronal compartments (Müller and Di Benedetto, 2025).

The third cross-cutting theme is methodological integration: multimodal imaging, AI, omics, and clinical phenotyping are transforming fragmented signals into system-level maps. Hewitt et al. review AI-enabled fusion of PET, MRS, MRI, and optical imaging, proposing three testable hypotheses: conserved coupling patterns distinguish healthy from pathological aging; excitatory–inhibitory reorganization reflects compensatory signaling rather than linear degeneration; and AI integration generates mechanistic predictors of cognitive, affective, and motor decline. They also emphasize that PET, MRS, MRI, and optical imaging each provide partial readouts, and that AI-driven harmonization is essential for cross-site reproducibility and mechanistic inference. The bibliometric studies by Chen et al. and Zhou et al. map the PD-related glutamate and GABA research landscapes, revealing how research hotspots have shifted toward α-synuclein, neuroinflammation, and anxiety. Al-Sheikh et al. provide a transcriptomic resource linking glial GABA to neuronal aging, while Liu et al. evaluate *in vivo* confocal microscopy and multimodal esthesiometry for structural–functional phenotyping in dry eye disease. Together, these approaches move the field from descriptive snapshots toward mechanism-informed, multiscale mapping of neurotransmitter systems (Kalpouzos and Persson, 2025).

Several cross-cutting conclusions emerge. First, excitatory–inhibitory imbalance is a shared substrate, with dopamine, glutamate, and GABA shaping network stability and vulnerability. Second, aging and disease progress from compensatory signaling to decompensation, where initially adaptive changes become maladaptive under sustained stress. Third, the neuro–immune–glial triad is central to amplifying dysfunction and shaping neurotransmitter availability. Fourth, the field is shifting from single-transmitter views to system-level frameworks enabled by multimodal imaging, AI, transcriptomics, and bibliometrics. Fifth, these advances inform precision phenotyping and nerve-directed therapies, from dopamine-modulating approaches in multiple sclerosis to glutamatergic–GABAergic interventions in Parkinson's disease and neuroprotective strategies targeting corneal nerve dysfunction in dry eye disease.

Despite these advances, critical gaps remain. Causal validation is limited, requiring longitudinal and interventional studies that link molecular changes to circuit dysfunction and clinical outcomes. Cross-species translation from nematode or rodent models to humans needs rigorous validation. Multimodal standardization is hindered by inter-site, inter-scanner, and inter-cohort variability; tools such as ComBat and Centiloid scaling offer partial solutions but cannot normalize all neurotransmitter modalities. AI models require external validation, explainability, uncertainty quantification, and fairness auditing before clinical deployment. Biomarker specificity remains an obstacle, as corneal nerve changes, CSF metabolites, and imaging signatures may reflect general neurodegeneration rather than disease-specific mechanisms. Finally, sex, age, comorbidities, and lifestyle factors powerfully shape neurotransmitter homeostasis yet are rarely integrated into mechanistic models.

Collectively, this Research Topic establishes a foundation for mechanism-informed, multimodal, precision-medicine-oriented research on neurotransmitter-related brain aging. Neurotransmitters should be regarded not as isolated molecules but as dynamic components of interconnected systems spanning synapses, glia, immune compartments, large-scale circuits, and behavior. By integrating molecular, imaging, computational, and clinical approaches, the field is advancing toward early risk stratification, mechanistic subtyping, and targeted interventions to mitigate age-related neurological decline. We thank all contributing authors, reviewers, and editorial board members for their efforts in assembling this Volume II Research Topic.
